# No change in the regional distribution of tidal volume during lateral posture in mechanically ventilated patients assessed by electrical impedance tomography

**DOI:** 10.1111/j.1475-097X.2010.00933.x

**Published:** 2010-07

**Authors:** Thomas Bein, Franz Ploner, Markus Ritzka, Michael Pfeifer, Hans J Schlitt, Bernhard M Graf

**Affiliations:** 1 Department of Anesthesiology, Regensburg University HospitalRegensburg, Germany; 2 Department of General Surgery, Regensburg University HospitalRegensburg, Germany; 3 Department of Anesthesiology, Hospital SterzingSterzing, South Tirolia, Italy; 4 Department of Pneumonology, Regensburg University HospitalRegensburg, Germany

**Keywords:** acute lung injury, electrical impedance tomography, lateral position, mechanical ventilation, tidal volume

## Abstract

We assessed the distribution of regional lung ventilation during moderate and steep lateral posture using electrical impedance tomography (EIT) in mechanically ventilated patients. Seven patients were placed on a kinetic treatment table. An elastic belt containing 16 electrodes was placed around the chest and was connected to the EIT device. Patients were moved to left and right lateral positions in a stepwise (10°) mode up to 60°. EIT images [arbitrary units (AU)] were generated and scanned for assessment of relative ventilation distribution changes [tidal volume (*V*_T_)]. A calibration procedure of arbitrary units (AUs) versus ventilator-derived *V*_T_ performed in all patients during three predefined positions (supine, 60°-left dependent and 60°-right-dependent) showed a significant correlation between *V*_T_ in supine, left and right lateral positions with the corresponding AUs (*r*^2^ = 0·356, *P*<0·05). Changes in *V*_T_ were calculated and compared to supine position, and specific regions of interest (ROIs) were analysed. In our study, in contrast to recent findings, a change in lateral positions did not induce a significant change in regional tidal volume distribution. In right lateral positions, a broader variation of *V*_T_ with a trend towards an increase in the dependently positioned lung was observed in comparison with supine. Lateral positioning promotes the redistribution of ventilation to the ventral regions of the lung. The use of EIT technology might become a helpful tool for understanding and guiding posture therapy in mechanically ventilated patients.

## Introduction

Changes in body position are known to influence respiratory mechanics and – in patients with acute respiratory disease or receiving mechanically ventilation – the pulmonary gas exchange. Positioning therapy by systematic changes in the body orientation [lateral position (Ibanez *et al.*, 1981), prone position or continuous ‘rotation’ using a special bed (Staudinger *et al.*, 2001)] is often used in patients suffering from respiratory failure as a measure to support artificial ventilation. By the application of various investigation techniques, the effects of steep lateral positioning on respiratory mechanics, the ratio of ventilation to perfusion, the pulmonary gas exchange, or the influence on hemodynamics have been examined (Nelson & Anderson, 1989; Schellongowski *et al.*, 2007). In a pilot study, Hedenstierna *et al.* (1984) studied the distribution of ventilation during anaesthesia in a two-compartment lung model in which each lung was ventilated separately by means of a double-lumen oro-tracheal tubus. Turning patients into lateral positions, it could be shown that the dynamic lung compliance was lower and the lung resistance was higher in the dependent lung than in the non-dependent lung. In children and adults suffering from respiratory disease, the distribution of perfusion (infusion of ^99m^Tc-macroaggregated albumin) and ventilation (inhalation of ^81m^Kr) was assessed (Bhyan *et al.*, 1989) while placing patients in supine and lateral positions. In these patients, it was found that in the lateral decubitus positions, the dependent lung received more of the total perfusion, while ventilation changed in the opposite direction. In thirty-four ventilated patients with respiratory failure, the effect of 90° lateral positioning on oxygenation, respiratory mechanics and hemodynamics was assessed (Thomas *et al.*, 2007). Dynamic compliance decreased during lateral positioning while arterial oxygenation was not affected. Furthermore, Schellongowski *et al.* (2007) investigated the effects of prolonged lateral steep position on respiratory mechanics, pulmonary gas exchange and hemodynamics. They placed the patients in a specially designed motor-driven bed, allowing a continuous lateral rotation up to 62°. In accordance with Thomas *et al.* (2007), they observed an impairment in the compliance of the respiratory system, but individual responses to position changes were unpredictable.

The aim of our present study was to assess the distribution of regional lung ventilation during lateral posture of patients suffering from acute lung injury (ALI), because those positioning manoeuvres are popular in intensive care units, but there is lack of data demonstrating the exact effects and elucidating the (patho-)physiologic mechanisms. For assessment, we used electrical impedance tomography (EIT) which is a non-invasive, radiation-free monitoring tool (Frerichs *et al.*, 2007) allowing the correct determination of changes in regional air content. We hypothesized that a stepwise change of lateral position will influence regional ventilation distribution in the dependent and non-dependent lungs.

## Patients and methods

The study was approved by our Institutional Review Board. After obtaining informed consent from patients relatives, seven patients with ALI were enrolled into the study. Characteristic data of the patients are presented in Table 1. In most patients, ALI was as a result of multiple trauma; and in all patients, bilateral infiltrates were radiologically identified. Patients with unilateral lung injury were excluded. Because of the recommendations of published studies (Pape *et al.*, 1994; Stiletto *et al.*, 2000) and following our clinical algorithm, the patients were placed in a special bed allowing a continuous lateral rotation up to 60° on each side (Rotorest, Kinetic Concepts, San Antonio, TX, USA) aimed at a prevention of posttraumatic pulmonary complications (ventilator-associated pneumonia) and/or an improvement in pulmonary gas exchange.

**Table 1:** Characteristic data of the patients.

Patient	Sex	Age (years)	Diagnosis	LIS	PaO_2_/FIO_2_	PEEP (cm H_2_O)	*V*_T_ (ml)
1	M	52	Pulmonary aspiration	2·5	192	14	490
2	M	53	Trauma	2·0	275	14	530
3	M	62	Multiple trauma	2·25	296	12	520
4	M	55	Multiple trauma	2·0	309	12	480
5	M	52	Multiple trauma	2·5	169	14	420
6	M	63	Multiple trauma	2·25	192	14	440
7	M	43	Trauma	2·0	266	14	350

LIS, Lung Injury Score (Murray: a score >2·5 indicates the presence of acute respiratory distress syndrome); PEEP, positive end expiratory pressure; *V*_T_,tidal volume.

Patients were ventilated in a pressure-controlled mode. Inspiratory pressure was titrated to receive a ‘lung protective’ tidal volume (*V*_T_ = 6 ml kg^−1^ predicted body weight), and inspiration/expiration ratio was set 1:1 in all patients. Ventilation frequency was set between 15 and 20 breaths per minute aimed at tolerating a moderate ‘permissive’ hypercapnia/acidosis (PaCO_2_ < 60 mmHg and/or pH > 7·25).

**Figure 1:**
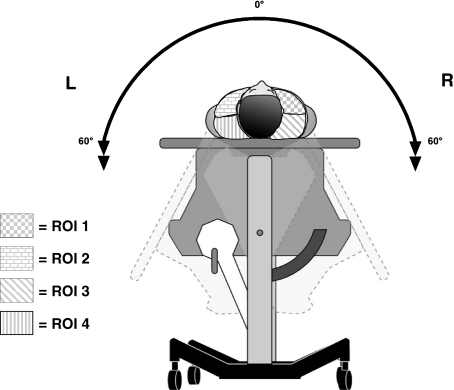
Patient placed on the kinetic treatment table allowing continuous or stepwise lateral positioning. Four regions of interest (ROIs) are marked. Note the view in a cranial-to-caudal axis. L = left, R = right.

After correct placement on the kinetic treatment table (Fig. 1), patients were moved in lateral positions in a stepwise (10°) mode. Left or right lateral posture was started in a randomized order. After movement to a 10°-step, patients were allowed to rest for 2 min and subsequently an EIT examination was performed. During the study period, ventilation parameters were not changed. During EIT measurement, the arterial oxygen saturation (SaO_2_), assessed by pulse oxymetry, mean arterial pressure (MAP) by indwelling arterial line and heart frequency (HF) were documented. For proper measurement of hemodynamic variables, the transducer system was fixed at the movable part of the kinetic bed close to the patient at the level of the left atrium. Furthermore, inspiratory plateau pressure (*p*_plat_) and tidal volume (*V*_T_) were noticed and quasi-static compliance (*c*_stat_) was ‘derived from the ventilator’s calculation referring to the whole lung (left and right)’ by using the formula 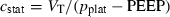, where *p*_plat_ was obtained after performing an inspiratory hold manoeuvre. Additionally, an end-expiratory hold manoeuvre was performed to exclude intrinsic positive end expiratory pressure (PEEP).

### Electrical impedance tomography technique and measurements

The principle of EIT is the calculation of regional ventilation distribution by the assessment of relative impedance changes in one predefined cross-sectional slice of the lung. An extensive description of the method can be found elsewhere (Frerichs *et al.*, 2003). For EIT measurements, we used an elastic belt, in which 16 electrodes were integrated. The belt was placed around the thorax shortly below the mammils (corresponding to *ca.* 5th intercostal space) and was then connected to the EIT device (EIT Evaluation Kit 2, Draeger, Lübeck, Germany). The electrodes were always placed in the same mode: 1st electrode at the left bound of sternum up to 16th electrode at the right sternum. The duration of each EIT recording was 2 min. During lateral positions, the correct placement of the belt was controlled frequently.

### Electrical impedance tomography calculation

From each measurement, functional EIT images were generated using the approach described by Victorino *et al.* (2004). For determination of relative ventilation distribution changes, an alternating current was injected between sequential pairs of adjacent electrodes. Voltages between adjacent pairs of non-injecting electrodes were measured during each current injection. After one complete rotation of the current injection, the resulting 208 measurements also called a ‘frame’ were used to reconstruct one single image, using a finite element method (FEM)-based linearized Newton-Raphson reconstruction algorithm (Zlochiver *et al.*, 2003). Subsequently, dynamic images representing relative impedance changes versus a baseline frame were generated at 20 frames per second. Within the initial EIT recording where patients were placed in supine position, the frame with the lowest summarized voltages was defined as the baseline frame. The pixel values of the images represented the percentage changes of local impedance versus the defined baseline. Such a procedure assumes that there is no information of absolute values of tissue impedance, but only on relative changes. A customized software automatically extracts pixel information from regions of interest (ROIs). In recent experimental and clinical investigations, it has been clearly demonstrated that these changes in pixels determine changes in regional air content/lung aeration (Hinz *et al.*, 2003; Riedel *et al.*, 2005; Wrigge *et al.*, 2008).

### Assessment of tidal volume and calibration

Tidal variations were assessed by the calculation of relative pixel changes between the end-expiratory and the end-inspiratory situation. These changes were expressed by ‘arbitrary units’ (AUs). We calculated the mean value of AUs during a 2-min measurement period including 30–40 values (= breaths per 2 min). We interpreted these changes in AUs as *tidal volume* (*V*_T_) variations. Additionally, we performed a calibration of AUs versus ventilator-derived tidal volumes in all patients during three predefined positions (supine, 60°-left dependent and 60°-right-dependent) by estimation of the correlation between both variables.

**Figure 3:**
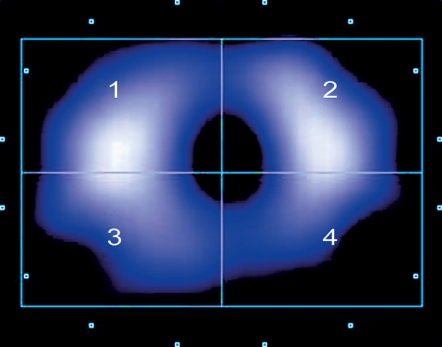
Original registration of an electrical impedance tomography (EIT) recording during supine position. Note the definition of four regions of interest (ROIs) from a caudal-to-cranial view.

Regions of interest (ROIs) were defined as following (Fig. 1 and 3) during assessment in supine position: right ventral part of the lung (ROI 1), left ventral part of the lung (ROI 2), right dorsal part of the lung (ROI 3), left dorsal part of the lung (ROI 4). Accordingly, during lateral positions, the ROIs experienced gravitational changes as follows: during right lateral position, ROI 1 and ROI 3 referred to dependent regions of the whole lung, while ROI 2 and ROI 4 were elevated, and vice versa in left lateral posture. Calculations of variations in *V*_T_ were performed in each ROI and additionally in right lung (ROI 1 + 3), left lung (ROI 2 + 4), ventral ‘near-sternum’-part of the whole lung (ROI 1 + 2) and dorsal ‘near-spine’-part of the whole lung (ROI 3 + 4), respectively.

### Statistical analysis

Statistical analysis was performed with SPSS, Version 16·0 (SPSS Inc., Chicago, IL, USA). Wilcoxon test was used to test the significance of changes in hemodynamics and AUs of *V*_T_ during positioning changes in comparison with supine. Pearsons correlation coefficient was calculated for the correlation between tidal volume and tidal variation. Results were considered significant at *P*<0·05.

## Results

### Calibration

**Figure 2:**
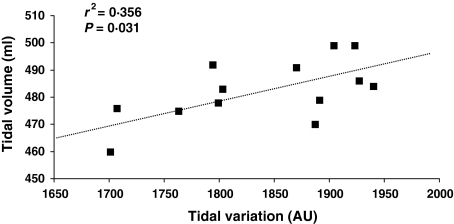
Calibration of arbitrary units versus ventilator-derived tidal volumes in all patients during three predefined positions (supine, 60°-left dependent and 60°-right-dependent) by estimation of the correlation between both variables.

The calibration procedure showed a significant correlation between *V*_T_ in supine, left and right lateral postures with the corresponding AUs [*r*^2^ = 0·356, *P*<0·05 (Fig. 2)].

### Haemodynamics and arterial oxygenation

The 10°-stepwise lateral positioning with the left lung down increased the MAP significantly (Table 2), but had no marked influence on HF or arterial oxygenation (SaO_2_). In right lateral position, MAP and SaO_2_ remained unchanged with a trend towards an increase in HF.

**Table 2:** Changes in mean arterial pressure (MAP), heart frequency (HF), and arterial oxygen saturation (SaO_2_) during lateral posture in seven patients (mean ± standard deviation, *P<0·05).

Position	MAP (mmHg)	HF (bpm)	SaO_2_ (%)
60° left	91 ± 22*	89 ± 9	97·9 ± 3·2
50° left	88 ± 19*	89 ± 9	98·0 ± 2·8
40° left	86 ± 18*	90 ± 8	97·6 ± 2·7
30° left	82 ± 15	89 ± 8	97·7 ± 3·0
20° left	81 ± 13	89 ± 8	97·9 ± 3·2
10° left	77 ± 12	86 ± 11	97·6 ± 3·2
***Supine***	***75 ± 10***	***89 ± 8***	***98***·***6 ± 1***·***6***
10° right	70 ± 8	93 ± 9	97·7 ± 2·8
20° right	71 ± 9	93 ± 10	97·9 ± 2·5
30° right	75 ± 13	92 ± 9	97·4 ± 2·9
40° right	76 ± 13	92 ± 9*	97·6 ± 2·8
50° right	76 ± 16	91 ± 10	97·4 ± 2·8
60° right	76 ± 16	90 ± 11	97·4 ± 2·8

Bold values given under ‘supine’ are the reference for other values

### Ventilation parameters

The ventilation mode (ventilation frequency, PEEP, inspiratory pressure, I:E ratio) was not changed during the study period. In stepwise *left* lateral position, a modest but significant increase in inspiratory plateau pressure (*p*_plat_) was observed in comparison with supine and a decrease in tidal volume (*V*_T_) during a 60° steep posture (Table 3). Quasi-static compliance was reduced statistically significant in such a positioning. In right lateral positioning, we found no marked changes in *p*_plat_ or *C*_stat_ but a trend towards an increase in *V*_T_.

**Table 3:** Changes in inspiratory plateau pressure (*p*_plat_), tidal volume (*V*_T_) and static compliance (*C*_stat_) during lateral posture in seven patients (mean ± standard deviation, *P<0·05). ‘All values are derived from the ventilators calculation and they refer to the whole lung (left and right)’.

Position	*p*_plat_ (cm H_2_O)	*V*_T_ (ml)	*C*_stat_ (ml cm^−1^ H_2_O)
60° left	26·1 ± 2*	460 ± 89*	38·7 ± 7*
50° left	25·6 ± 2	476 ± 92	41·1 ± 7*
40° left	25·0 ± 1	475 ± 92	43·1 ± 8
30° left	24·9 ± 2	492 ± 83	44·0 ± 8
20° left	24·7 ± 2	483 ± 83	44·1 ± 9
10° left	24·4 ± 2	478 ± 87	42·3 ± 7
***Supine***	***24***·***6 ± 3***	***479 ± 96***	***44***·***0 ± 7***
10° right	25·1 ± 2	491 ± 86	42·1 ± 10
20° right	25·3 ± 2	499 ± 79	43·4 ± 10
30° right	25·1 ± 2	486 ± 80	42·0 ± 7*
40° right	25·1 ± 2	499 ± 74	44·9 ± 9
50° right	25·6 ± 1	484 ± 81	40·9 ± 6
60° right	25·0 ± 2	491 ± 82	42·0 ± 8

Bold values given under ‘supine’ are the reference for other values

### Tidal volume variation and distribution

**Figure 4:**
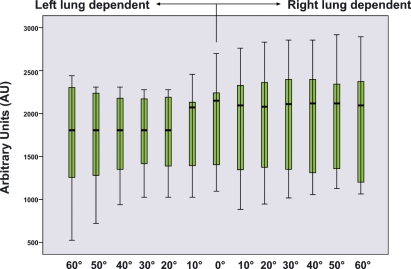
Changes in arbitrary units (AUs) as surrogate parameters for tidal volume variations during lateral posture. Box and whisker plots (95th, 75th, 50th, 25th, 5th percentiles).

In lateral positions, a wide variation in tidal volume levels – expressed by AUs – was observed. Especially, in right lateral positions (Fig. 4), such a variation was noticed. Turning the patients in steep right lateral positions resulted in a trend towards an increase in tidal volume, while the median values remained unchanged. In left lateral positions, a different pattern of changes was found: turning the patients left laterally resulted in a tendency towards a reduction in tidal volume, but no significant changes in both lateral postures because of the broad variation of tidal volume variations were found.

**Figure 5:**
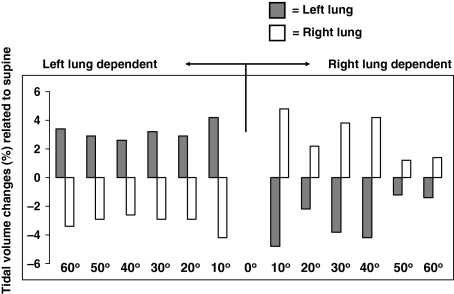
Relative changes (%) in tidal volume distribution of the left lung (ROI 2 + 4) and the right lung (ROI 1 + 3) during lateral posture in comparison with supine.

Additionally, we analysed the changes in tidal volume distribution of the *left lung* (ROIs 2 + 4) and *right lung* (ROIs 1 + 3) separately (Fig. 5). In left lateral positions, no marked changes (≈4%) in *V*_T_ of the left lung were noticed, while in right lateral positions, a modest increase in the tidal volume of the right lung, especially in 30–40° positions, was found: in our patients, the lateral position resulted in a trend to increase in *V*_T_ in the down-positioned lung.

**Figure 6:**
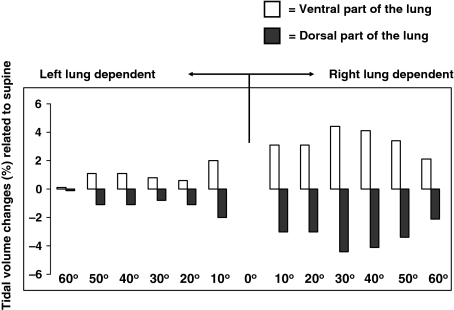
Relative changes (%) in tidal volume distribution of the ventral part (‘near-sternum’-regions [ROIs 1 + 2]) and the dorsal part of the lung (‘near-spine’-regions [ROIs 3 + 4]) in a vertical axis view during lateral posture in comparison with supine.

The analysis of changes in tidal volume distribution of the ventral part of the lung (‘near-sternum’-regions [ROIs 1 + 2]) and the dorsal part of the lung (‘near-spine’-regions [ROIs 3 + 4]) in a vertical axis view is demonstrated in Fig. 6. Lateral positioning promotes the redistribution of ventilation to the ventral regions of the lung. Such a trend is more pronounced while turning the right lung down in comparison with left lateral posture.

## Discussion

The aim of our study was to assess the effects of stepwise lateral positioning on the distribution of regional ventilation in mechanically ventilated patients suffering from ALI. For such a purpose, we used the technique of EIT which allows the determination of changes in regional air content by generation of electrical impedance changes.

In a recent validation study, Victorino *et al.* (2004) demonstrated a reliable assessment of ventilation distribution during mechanical ventilation by EIT. They compared EIT-findings with dynamic computerized tomography in a heterogeneous population of critically ill mechanically ventilated patients. They observed acceptable agreements between both technologies in detecting right–left ventilation imbalances or the relative distribution of ventilation. Numerous other clinical and experimental investigations have studied the EIT-technique in healthy volunteers or animals or in critically ill situations (Putensen *et al.*, 2007). Wrigge *et al.* (2008) performed an experimental study aimed at determining the validity of EIT to monitor regional ventilation distribution in lung injury. In lung injured pigs, in which the induction of lung failure was performed either by acid aspiration (direct injury) or by abdominal hypertension (indirect injury), they found an accurate assessment of regional ventilation distribution or recruitment by EIT. The effect of PEEP variation on regional lung recruitment and derecruitment was assessed using EIT by Meier *et al.* (2008), and they found EIT suitable for monitoring the dynamic effects of PEEP variations on the regional change of tidal volume.

To our knowledge, no data exist assessing the effects of systematic changes of the patients body position on regional lung ventilation under the conditions of lung injury and mechanical ventilation. Positioning therapy (lateral position, prone position, continuous lateral rotation) is an attractive adjunctive option in patients suffering from ALI. We were interested in assessing the exact effects of these positioning manoeuvres, because the physiologic mechanisms often remain unclear.

We investigated the effects of lateral positioning in seven patients with ALI, and the main results of our study are summarized as follows:

In our study, a change in lateral positions did not induce a significant change in regional tidal volume distribution. In right lateral positions, a broader variation of *V*_T_ with a trend towards an increase in the dependently positioned lung was observed in comparison with supine. Lateral positioning promotes the redistribution of ventilation to the ventral regions of the lung.

Comparing the analysis of our data with the existing literature, some corresponding and some differing findings are seen: in accordance with our results, a reduction in static compliance in lateral positions compared to supine has been stated in healthy volunteers (Baydur *et al.*, 1996) as well as in mechanically ventilated patients (Schellongowski *et al.*, 2007; Thomas *et al.*, 2007). Furthermore, we saw a tendency for an increase in *V*_T_ in the *dependent* lung, which is in contrast to results from other studies (Amis *et al.*, 1984; Hedenstierna *et al.*, 1984; Hoffman, 1985; Baydur *et al.*, 1996): in ventilated patients with a PEEP of 9 cm H_2_O, Hedenstierna *et al.* (1984) found a slight increase in *V*_T_ in the *elevated* lung and Amis *et al.* (1984) observed a decrease in *V*_T_ in the dependent lung in healthy male volunteers. There is a lack of exact explanations to the differences of our findings to previous works. On the one hand, we have used a new technique which might allow a more precise view into pulmonary physiological mechanisms. On the other hand, no experimental investigation comparing EIT to reference imaging techniques exists because of some severe technical problems associated with lateral posture (adequate animal model, realization of computed tomography with positional changes, etc.).

Interestingly, we noticed a wider variation of tidal volume distribution in steep right lateral position in comparison with left lateral position. Because of the anatomy of the mediastinal organs, the human chest is filled in an asymmetric pattern; and during lateral positioning, some gravitational effects may be active. Although heart–mediastinum–lung interactions play an important role in the physiological understanding of mechanical ventilation, there is a lack of experimental or clinical data investigating the contribution of such gravitational effects of the heart and mediastinum during posture changes in ventilated patients. In a recent case report, a severe hypoxia was reported in a 87-year-old woman during right lateral position for hip arthroplasty surgery and chest X-rays taken in lateral posture and in supine revealed that the right lung volume was decreased remarkably because of the extreme downward shift of the mediastinum during right decubitus position (Yasuda *et al.*, 2007). We investigated the effects of extreme lateral posture (62°) on hemodynamics and plasma atrial natriuretic peptide levels in critically ill, ventilated patients (Bein *et al.*, 1996); and we observed a significant increase in cardiac index, intrathoracic blood volume, right ventricular end-diastolic volume and serum atrial natriuretic peptide levels in the left lateral position in comparison with supine. In echocardiographic controls, an increase in right ventricular end-diastolic diameters in the left dependent position and shortened diameters in the right dependent position was found. We hypothesized that these findings are because of altered distensibility of the right ventricle probably caused by regional intrathoracic gravitational changes. Additionally, interpreting the results from our present study, the hypothesis might be added that the variation of *V*_T_, predominantly during right lateral posture, analogously with regional intrathoracic gravitational changes might predominantly act in the ventral part of the lung (Fig. 6).

Our study has some methodic limitations and therefore the results have to be interpreted with caution: (i) we do not know what happens with the content of the chest during lateral posture, because there are no reference imaging techniques (dynamic magnetic resonance technique or computerized tomography) during lateral posture available. We cannot exclude that our measurements not only reflect ‘true’ changes in ventilation but additionally a movement of intrathoracic organs. (ii) Patients were ventilated in a pressure controlled mode with a relatively high PEEP (≥12 cm H_2_O), which might have influenced or ‘uniformed’ the distribution of ventilation during lateral posture. It has been shown by Hedenstierna *et al.* (1984) that the application of zero PEEP (ZEEP) reinforced the uneven ventilation distribution between lungs markedly. As a result, the findings of clinical investigations referring to spontaneously breathing subjects are not comparable to findings from artificially ventilated patients with positive pressure and PEEP. (iii) The inspiratory flow is an important variable influencing the distribution of inspired gas (Rehder *et al.*, 1981). The inspired flow in the ventilators mode was not varied in our study; and on the other hand, inspiratory flow was not measured exactly at the inspiratory part of the ventilation system. Thus, we cannot exclude that posture-related changes in the inspiratory flow might have influenced the ventilation distribution pattern.

In summary, in this pilot study, the innovative technique of EIT was used to assess ventilation distribution changes during lateral postures in mechanically ventilated patients and we found some data, which might help to understand the physiologic basis of systematic body position changes in intensive care. The data encourage us to use EIT in further trials on positioning therapy to better elucidate the mechanisms of such measures and to identify patients, who might benefit from these adjunctive strategies.
